# Occupational Injuries in Greece: A Systematic Review of the Literature

**DOI:** 10.7759/cureus.77661

**Published:** 2025-01-19

**Authors:** Pantelis Politis, Michalis Leotsinidis, Eleni Jelastopulu, Irini Tatani

**Affiliations:** 1 Department of Public Health, Medical School, University of Patras, Patras, GRC; 2 Orthopedic Department, Medical School, University of Patras, Patras, GRC

**Keywords:** accidents, agricultural, greece, occupational, work-related injury

## Abstract

Occupational injuries represent a significant public health issue, impacting workers' health, productivity, and economic stability. This systematic review aims to summarize and analyze the existing literature on occupational injuries in Greece, focusing on their prevalence, types, causes, and associated risk factors. A systematic search was performed using the databases PubMed, Scopus, and Web of Science. Keywords used included ("occupational" OR "agricultural" OR "work-related") AND ("injuries" OR "accidents") AND ("Greece" OR "Greek"). Inclusion criteria were clinical studies providing quantitative data on occupational injuries in Greece. Case reports, reviews, studies in other than English language, and conference papers were excluded. Data were extracted regarding study design, population, types of injuries, causes, and risk factors. A total of 22 studies met the inclusion criteria, encompassing various sectors such as construction, agriculture, industry, and healthcare. Most studies were cross-sectional and retrospective cohorts. The prevalence of occupational injuries varied widely across different sectors, with construction and agriculture showing the highest rates, reaching 30%. Common types of injuries included fractures, sprains, and cuts, with machinery-related accidents and falls being the most frequent causes. Risk factors identified included young age, working inexperience, lack of safety training, insufficient protective equipment, and poor regulatory enforcement. Occupational injuries in Greece are a prevalent and diverse problem, heavily influenced by sector-specific factors and general safety culture. There is a need for improved regulatory frameworks, enhanced safety training programs, and better enforcement of existing laws to reduce the incidence of these injuries.

## Introduction and background

Occupational injuries refer to physical harm or damage that occurs to individuals while performing their job duties, ranging from minor cuts and bruises to severe injuries such as fractures, amputations, or even fatalities. The prevalence of occupational injuries varies by industry, job role, and geographical region. High-risk industries include construction, agriculture, manufacturing, and mining. Data from different countries often reveal patterns specific to local economic activities and regulatory environments [1,2].

The causes of occupational injuries are diverse. Human factors such as lack of training, working inexperience, use of protective equipment, human error, and fatigue play a significant role [3]. Environmental factors include unsafe working conditions, harsh weather, poor lighting, and noise pollution. Mechanical factors involve defective machinery or improper use of tools. Organizational factors cover inadequate safety policies, lack of supervision, and high workload and pressure from supervisors [4,5].

The impact of occupational injuries is significant. Financially, they lead to direct costs like medical expenses and compensation claims, as well as indirect costs such as lost productivity and the need to train replacement workers. Socially, these injuries affect workers' families and communities and can result in long-term disability and reduced quality of life. Psychologically, injured workers may experience mental health issues like post-traumatic stress disorder, depression, and anxiety [6].

Occupational injuries in Greece have several distinct characteristics shaped by the country's economic structure, regulatory environment, and cultural factors. The prolonged economic crisis in Greece has led to cost-cutting measures that often affect workplace safety, as companies may prioritize financial survival over investing in safety training and equipment, resulting in an increase in workplace injuries [7-9]. A significant portion of the workforce is employed in informal or semi-formal sectors where labor laws are not strictly followed, leading to underreporting of injuries and lack of access to proper safety measures and healthcare. Immigrant workers, who constitute a large part of the informal labor force, are particularly vulnerable due to language barriers, lack of training, and exploitation. There is often a cultural attitude of fatalism and acceptance of risks in certain sectors, especially among older workers, leading to complacency about safety measures and reluctance to report injuries [10]. Additionally, family-run businesses, which are common in Greece, may not adhere strictly to formal safety protocols, relying instead on traditional practices that may not meet modern safety standards [11].

In Greece, the issue of workplace safety has been highlighted by several studies, but a comprehensive understanding of the current situation requires a systematic review of existing literature. This systematic review aims to synthesize the available evidence on the prevalence, types, causes, and risk factors associated with occupational injuries in Greece.

## Review

Materials and methods

A systematic search was conducted using the databases PubMed, Scopus, and Web of Science, following the Preferred Reporting Items for Systematic Reviews and Meta-Analyses (PRISMA) guidelines and utilizing EndNote X3 software (Thomson Reuters) [12]. Keywords used included ("occupational" OR "agricultural" OR "work-related") AND ("injuries" OR "accidents") AND ("Greece" OR "Greek"). Inclusion criteria were clinical studies providing quantitative data on occupational injuries in Greece. Case reports, reviews, studies in other than English language, and conference papers were excluded. Moreover, studies focusing on chronic musculoskeletal occupational disorders and not acute trauma were also excluded. Data were extracted regarding study design, population, types of injuries, causes, and risk factors.

Results

**Figure 1:**
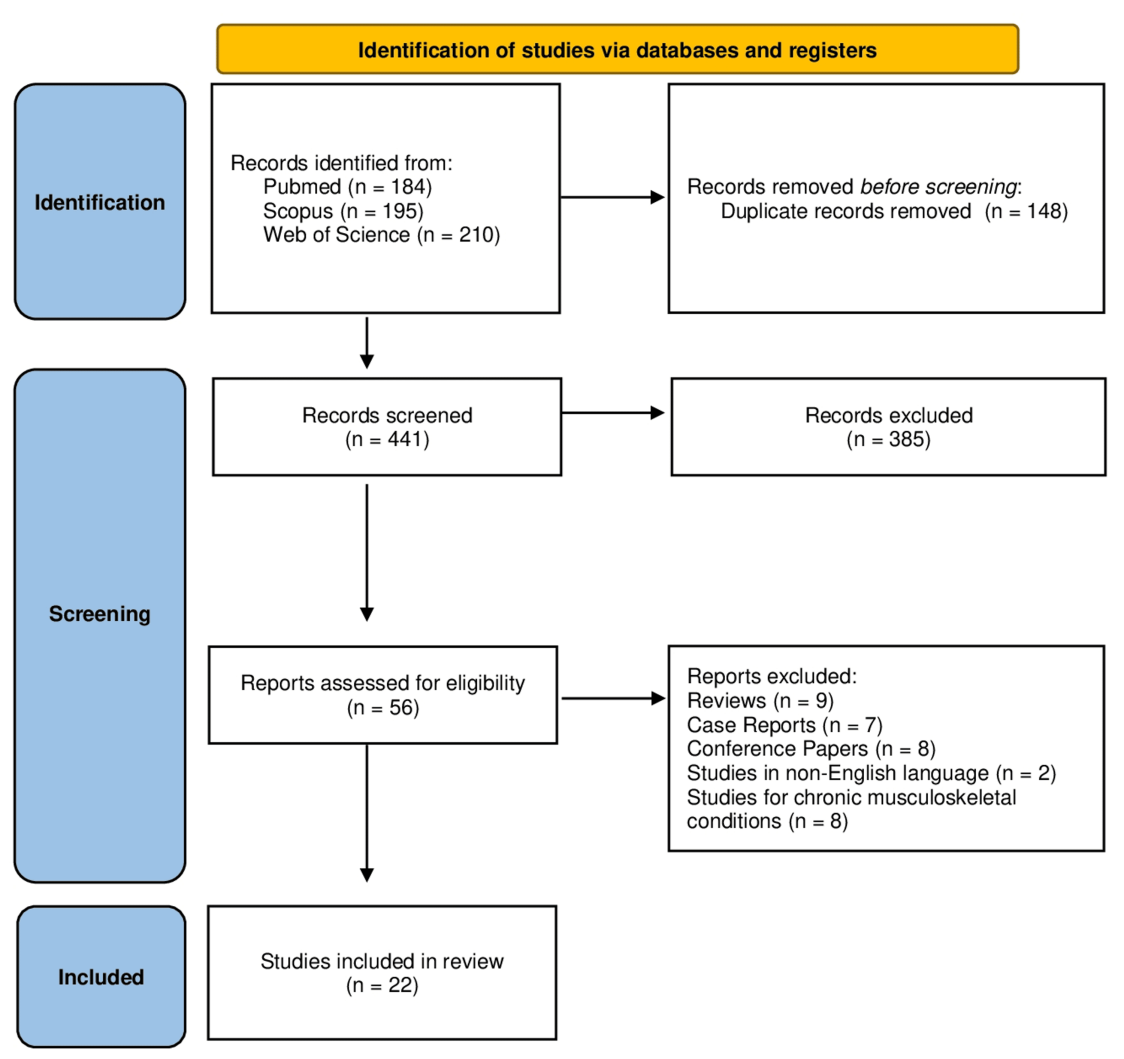
PRISMA study selection flowchart PRISMA: Preferred Reporting Items for Systematic Reviews and Meta-Analyses

Initially, 589 studies were identified after the primary search of online databases Pubmed, Scopus, and Web of Science. After checking for duplicate entries, 148 articles were excluded. Among the remaining 441 studies, 385 were rejected as irrelevant to the purpose of the study. Among the remaining studies, nine review papers, seven case reports, eight conference papers, two non-English studies, and eight studies about chronic musculoskeletal disorders were excluded. Finally, 22 studies remained for analysis (Figure 1).

The 22 included studies encompassed various sectors such as construction, agriculture, manufacturing, and healthcare. The study designs were predominantly cross-sectional and retrospective cohorts, based on medical records and self-reports, suggesting a high risk for information bias.

Injuries in the Agricultural Sector

Agricultural injuries are relatively common in Greece, with varying prevalence depending on the specific agricultural activities, regions, and seasons. In the olive oil industry, Politis et al. found a 30% annual prevalence of agricultural injuries [13]. In 2003, Alexe et al. investigated the characteristics of farm injuries in Greece for a five-year period by using a public database. After analyzing more than 4300 injuries, the authors found that the most common mechanism of occupational farm injuries among Greek nationals was falls on the same level [14]. Most farm-related injuries occurred in males with an age higher than 50 years old [13,14]. In olive oil gathering, work experience, diabetes mellitus, poor weather conditions, climbing habits, and non-use of protective gloves were definite risk factors for work-related injuries. The use of machinery like tractors, harvesters, and other heavy equipment posed significant risks [13]. The majority of farm-related injuries were contusions followed by fractures and open wounds [13,14]. Migrant workers usually sustained accidents due to falls from high levels, leading to multiple severe injuries, as well as snake and insect bites affecting upper limbs [14].

Equestrian-related injuries among Greek farmers are a significant concern due to the frequent use of horses in agricultural activities. Common types of equestrian-related injuries include falls from horses, kicks, bites, and crush injuries. Risk factors include lack of training and experience, improper use of equipment, such as saddles and bridles, and inadequate use of protective gear. According to a prospective study by Petridou et al., the estimated countrywide injury incidence for equestrian-related injuries was 21 per 100,000 person-years. The majority of injuries affected men. Fractures accounted for more than 30% of injuries in farming; head injuries accounted for approximately 50% of injuries among farmers. Farming injuries were more serious, with 25% requiring hospitalization. Spurs were the main causative factor for ankle fractures and dislocations [15].

Injuries in the Construction Sector

Three studies have analyzed occupational injuries in the construction sector in Greece, including onshore wind farms, construction projects in northern Greece, and construction buildings for the 2004 Olympic Games [16-18].

Occupational risks in onshore wind farms in Greece encompass a variety of hazards. Technicians frequently work at significant heights, often climbing turbines that are 70-120 meters tall, in confined spaces. They are often exposed to harsh weather conditions and sustain risks associated with the mechanical components and electrical systems of turbines. According to Aneziris et al., during construction, fitters have the highest fatality risk, followed by crane operators. During maintenance, the highest fatality risk involves electrical/mechanical maintenance operators. The most common mechanisms of injury were falls from elevation and crush injuries from falling objects [16].

A retrospective study by Betsis et al. investigated work accidents in construction projects in northern Greece, between 2003 and 2007. Most accidents occurred in male unskilled workers of ages 24-44 years, with work experience of less than one year. Most injuries occurred in the summer and morning. The most common mechanism of accidents was falls and the most common type of injury was a fracture. Mortality of this type of injury was calculated at 13% [17].

During the lead-up to the 2004 Olympics, the construction sector in Greece experienced a notable number of occupational injuries. The urgency to complete facilities on time for the Olympics led to extended working hours and insufficient rest periods for workers, leading to a higher incidence of accidents. Safety protocols were often overlooked and proper training and safety equipment were sometimes neglected. In the period between 1999 and 2003, 63 fatal occupational injuries took place in East Attica, Greece. Most fatal injuries included falls from heights (58.7%), electric shocks (12.7%), and being crushed into or between objects (11.1%). Lack of protective equipment, task difficulty, and pressure from supervisors were identified as causative factors [18].

Injuries in the Power Industry

Workers in the power industry face risks from exposure to high-voltage electricity, which can lead to severe injuries or fatalities. Contact with moving machinery, heavy lifting, and working at heights also contribute to the risk of injuries. Moreover, the presence of hazardous chemicals used in power generation and maintenance can result in chemical burns, respiratory issues, and other health problems. In 2002, Batra and Ioannides assessed all electrocutions that occurred in the period between 1992 and 1996 to workers in the power industry of Greece. Researchers recorded 112 occupational electrocutions (22.4 per year), with a 6.25% mortality. Most of the electric accidents happened in the distribution sector (78.6%), followed by the production sector and the mines (9.8%). Most accidents affected network electricians (38.4%). About 74.1% of all electric accidents occurred to electricians aged 31-45 years. About 73.2% of electric accidents happened to electricians in their first 15 years of employment. The morning shift (09.00-15.00) was most commonly affected. Regarding the type of injury, electrocutions were the most common (57.2%), followed by burns (30.4%). The most common causes of electric accidents were a partial lack of protective equipment and measures (22.5%), wrong working method (18.8%), and failure of material or mechanical equipment (10.0%) [19].

Injuries in the Petrochemical Industry

Due to its hazardous working conditions, the petrochemical industry in Greece experiences a high rate of occupational injuries. Workers are exposed to high-risk situations involving heavy machinery, high-pressure systems, and extreme temperatures. Handling hazardous chemicals and the potential for explosions and fires are critical risk factors in the petrochemical industry. Common injuries result from contact with hot surfaces, falls, and machinery malfunctions [20].

Two studies have analyzed occupational injuries in the petrochemical industry in Greece [20,21]. Konstandinidou et al. published an analysis of more than 1100 reported incidents in the Greek petrochemical industry between 1997 and 2003. Most accidents occurred in the autumn and morning (09.00-14.00). The most common substances involved in incidents were gasoline and light hydrocarbons. The majority of accidents took place in the distillation unit and were attributed to human error (46%). Mortality was calculated as less than 0.3% [20,21].

Injuries in the Military Sector

Injuries among military personnel are relatively common due to the physically demanding nature of military training and operations. High physical demands during training and operations are a primary risk factor. Activities such as jumping from aircraft, intense physical training, and carrying heavy equipment contribute significantly to injury rates. Harsh environmental conditions, including extreme temperatures and rough terrains, increase the risk of injuries. The use of heavy and sometimes cumbersome equipment, including protective gear and weaponry, can contribute to both acute and chronic injuries [22,23].

Among 253 cadets receiving a seven-week basic combat training, occupational musculoskeletal injuries were associated with older age, female gender, high body fat percentage, and Greek nationality. Researchers concluded that overfat cadets had a 20% higher possibility of suffering an occupational injury [22]. Among 250 militants from Evros County, conscripted soldiers and professional soldiers had a 2-4 times higher risk of occupational injuries in comparison to army officers. The degree of risk perception due to electrical hazards, lighting, falls, and insufficient safety signs were associated with increased occurrence of accidents. The risk of occupational accidents was associated with age, time of service in the present unit, specialty, level of education, years of service, and overall military training [23].

Acoustic trauma and noise-induced hearing loss are common among military personnel due to their frequent exposure to high levels of noise from firearms, explosives, and heavy machinery. Among 39 young soldiers hospitalized for hearing loss after exposure to weapon impulse noise, “the most significant differences in pure-tone thresholds on initial testing were found in the frequency range 0.25-11.2 kHz, and especially in the 4-8 kHz region” [24]. Following treatment, a reduction in thresholds across most frequencies was observed, although partial recovery was achieved in most cases [24].

Injuries Among Firefighters

Firefighters in Greece face a high prevalence of occupational injuries due to the demanding nature of their job. Firefighters engage in physically intense activities, such as carrying heavy equipment, which increases the risk of musculoskeletal injuries. They are regularly exposed to extreme heat, smoke, toxic chemicals, and unpredictable structural collapses, contributing to various health risks. Among 3289 professional firefighters, with a mean age of 36.4 years, the rate of work-related injuries was 11%. Common injuries included burns, fractures, ankle sprains, and strains, even though acute low back pain was most commonly reported [25].

Injuries Among Healthcare Personnel

Healthcare workers in Greece face a high prevalence of occupational injuries, particularly needle-stick injuries, which sometimes are underreported. An older study, in 1999, analyzed the conditions of needle-stick and sharp injuries in a Greek general hospital in the period between 1990 and 1996. A total of 284 needle-stick and sharp injuries were reported by 247 healthcare workers, representing an overall rate of 2.4% reported injuries per 100 healthcare workers per year. Female nurses sustained the highest rates of accidents. About 61% of injuries were attributed to needles. The most common causes of injury were resheathing of used needles and garbage collection. About 49% of the injuries were reported in the patient's room, 22% in the operation theatre, 13% in the intensive care unit, 10% in laboratories, and 6% in the outpatient department. The majority of incidents occurred during the morning shift, and young workers (21-30 years old) were most commonly affected [26].

Falagas et al. prospectively studied the percutaneous exposure incidents of employees in a newly founded tertiary hospital in Athens, Greece, between 2002 and 2005. During the study period, 60 needle-stick and 11 sharp injuries were reported affecting nurses and members of the cleaning staff. The overall incidence of percutaneous injuries per 100 full-time employment years was 3.38 [27]. A cohort study by Samarkos et al. prospectively collected data on all percutaneous exposures reported in the period between 2008 and 2010 in a 950-bed tertiary care general hospital. A total of 374 percutaneous exposures were recorded, and the incidence rate was calculated at 23.1 incidents per 100 occupied beds per year. Nursing students were most commonly affected. The most common causes of injury were inappropriate sharps disposal (19%) and recapping (18%). The majority of incidents occurred during the morning shift [28].

A recent survey by Patsopoulou et al. investigated the prevalence, risk factors, and special characteristics of sharp injuries in 457 healthcare workers (75% women) in five public hospitals in Thessaly (central Greece). Results showed that 74.1% of the participants suffered from at least one injury. About 65% of injuries occurred in nursing and 62% of injuries took place during the morning shift. About 33% of the injuries were reported in the patient's rooms, 12% in the nurses' station, 10% in the emergency department, 9% in the intensive care unit, 8.4% in blood sampling, 8.4% in surgery, and only 7.8% in laboratories or other places. The overwhelming majority (96%) of sharp injuries affected hands. However, occupational injuries were reported in only 70% of accidents, and 53% of workers did not apply the suggested procedures and guidelines. The risk of sharp injuries was associated with age, level of education, shifts, and possibly sex [29].

Occupational injuries among Greek endodontists have been a subject of study due to the unique risks associated with dental practice. Needle-stick injuries are a common occupational hazard among Greek endodontists. Ocular injuries can occur from splashes of chemicals, bodily fluids, or debris during dental procedures. Two studies have assessed the special characteristics of occupational injuries among Greek endodontists [30,31]. The reported incidence of ocular accidents was 73%. Amalgam and sodium hypochlorite were the most frequently reported foreign bodies. About 16% of injured endodontists sought medical assistance. No permanent ocular damage was reported. Regular use of magnification was associated with a 70% decrease in injury risk, and years of clinical experience were associated with an 80% decrease in injury risk [30]. The reported incidence of percutaneous injuries was estimated at 1.35 per endodontist per year. Fingers were most commonly injured (75%). About 36% of injured endodontists sought medical assistance. The practice of four-handed endodontics was associated with a reduced number of percutaneous injuries (p=0.007); the performance of surgical endodontics increased their incidence (p=0.007) [31].

Injuries in the Restaurant Sector

According to a study by Carayanni et al., the reported rate of occupational injuries among restaurant employees was 44.3%. Risk factors for work-related injuries included conflicts with supervisors and colleagues, permanent stress and body pains, being kitchen staff, and lifting heavy loads [32].

Injuries in the Fishing Industry

Occupational injuries among Greek fishermen are a significant concern due to the hazardous nature of the fishing industry. Common types of injuries include falls overboard, slips, trips and falls, striking injuries, cuts, and lacerations. Greek laws and regulations govern maritime safety and the working conditions of fishermen. Compliance with these regulations is crucial for minimizing occupational hazards [33,34]. According to Frantzeskou et al., 28% of fishermen had experienced at least one injury, while 14% of them had a near-drowning experience. About 88% of fishermen worked in coastal fisheries and 73% used small fishing vessels. Overweight fishermen with cardiovascular incidents and dermatological, musculoskeletal, respiratory, hearing, stress, and anxiety problems possessed the greatest risk of occupational accidents. Use of alcohol, smoking, fatty food consumption, and lack of physical exercise also contributed to the increase in occupational injuries [33,34].

The findings of all the aforementioned studies are summarized in Table 1.

**Table 1:** Summary of the included studies AHEPA: American Hellenic Educational Progressive Association; NM: Not mentioned

Study	n	Source	Occupation	Most Common Injuries	Most Commonly injured Site	Risk Factors
Politis et al., 2023 [13]	166	Questionnaire (Achaia, Greece)	Olive workers	Contusions, fractures, open wound	Abdominal area, thoracic wall, head	Male gender, age >50 years, work experience >24 years, arterial hypertension, diabetes mellitus, climbing habits, non-use of protective gloves
Alexe et al., 2003 [14]	4326	Emergency Department Injury Surveillance System	Farm workers	Contusions, open wounds, fractures, sprains	Upper limbs, lower limbs, trunk	Male gender, age >65 years, winter
Petridou et al., 2004 [15]	140823	Emergency Department Injury Surveillance System	Equestrians	Contusions, open wounds, fractures	Neck, head, upper limbs	Male gender, age 55-74 years
Aneziris et al., 2014 [16]	19	Questionnaire (Greek Wind Farm, 16 wind turbines)	Wind farm workers	Falls from elevation, contact with falling objects, electrocution	NM	Fitters, crane operators, electricians
Betsis et al., 2019 [17]	413	Greek Work Inspection Organization	Construction workers	Fractures, contusions, bruises	Lower limbs, upper limbs, head	Male gender, age 24-44 years, work experience <12 months
Katsakiori et al., 2008 [18]	63	Center for Prevention of Occupational Risk (1999-2003)	Construction workers for the Olympic Games	Falls from a height, electrocution, crushing injury	NM	Lack of protective equipment, task difficulty, pressure from supervisor
Batra et al., 2002 [19]	112	Department of Safety at Work (1992-1996)	Electric power workers (only electric accidents)	Electrocution, burns	Upper limbs	Age 31-45 years, distribution sector, network electricians, work experience <15 years, lack of protective equipment, wrong working method, equipment failure
Konstandinidou et al., 2006 [21]	1115	Questionnaire (1997-2003)	Petrochemical sector workers	NM	NM	Distillation unit, human error, autumn/morning shift
Konstandinidou et al., 2011 [20]	1115	Questionnaire (1997-2003)	Petrochemical sector workers	NM	NM	Distillation unit, human error, autumn/morning shift
Havenetidis et al., 2011 [22]	290	Database recorded by physicians (7-week basic training)	Cadets	NM	NM	Female gender, older age, high body fat percentage, Greek nationality
Malliarou et al., 2012 [23]	250	Questionnaire (Evros County)	Militants	NM	NM	Age, time of service in the present unit, specialty, level of education, years of service, overall military training
Balatsouras et al., 2005 [24]	39	Database recorded by physicians	Soldiers hospitalized for hearing loss	Hearing loss in the frequency range 0.25-11.2 kHz	NM	NM
Katsavouni et al., 2015 [25]	3289	Questionnaire	Firefighters	Burns, fractures, sprains	NM	Impaired vision, difficulty in breathing, weightlifting, post-traumatic distress syndrome, chronic musculoskeletal disorder
Pournaras et al., 1999 [26]	247	Infectious control team (AHEPA Hospital)	Healthcare workers	Needle-stick injuries, scalpel injuries	Upper limbs	Female gender, age 21-30 years, resheathing of used needles, garbage collection
Falagas et al., 2007 [27]	73	Infectious control team (Henry Dunant Hospital)	Healthcare workers	Needle-stick injuries, sharp injuries	Upper limbs	NM
Samarkos et al., 2014 [28]	374	Infectious control team (Evaggelismos Hospital)	Healthcare workers	Percutaneous injuries	Upper limbs	Inappropriate sharps disposal, inappropriate scalpel recapping, nursing student
Patsopoulou et al., 2022 [29]	457	Questionnaire	Healthcare workers	Needle-stick injuries, sharp injuries	Hands	Age 18-25 years, work experience <10 years, nursing students
Zarra et al., 2013 [30]	147	Questionnaire	Endodontists (ocular accidents)	Chemical burn, foreign body	Eye	Male gender, regular use of magnification, clinical experience <20 years
Zarra et al., 2013 [31]	147	Questionnaire	Endodontists (percutaneous injuries)	Needle-stick injuries, sharp injuries	Fingers	Four-handed endodontics, no use of surgical endodontics
Carayanni et al., 2011 [32]	180	Questionnaire	Restaurant workers	Falls, slips	NM	Conflicts with supervisors and colleagues, permanent stress and body pains, being kitchen staff, lifting heavy loads
Frantzeskou et al., 2012 [33]	100	Questionnaire/Telephone interviews	Fish workers	Bites, soft tissue injuries	Hands, spine, knee	Small working area, use of unsuitable equipment, extreme weather conditions, exposure to solar radiation, stress
Frantzeskou et al., 2016 [34]	173	Questionnaire	Fish workers	NM	Fingers	Insufficient light, extreme weather conditions, poor infrastructure, extreme musculoskeletal loading

Discussion

The findings of this study indicate a high prevalence of occupational injuries in Greece, particularly in high-risk sectors like construction and agriculture. To the best of our knowledge, this is the first study summarizing the prevalence, types, risk factors, and special characteristics of occupational injuries in Greece. The common types and causes of injuries highlight the need for targeted interventions to improve workplace safety.

Occupational injuries in Greece encompass a wide range of incidents, from minor accidents to severe, life-threatening injuries. The prevalence rates vary significantly, with construction and agriculture showing higher rates of injuries. These industries are traditionally associated with higher physical risks, involving machinery, heights, and hazardous materials [13,14,17,35]. The prevalence of occupational injuries in Greece is influenced by several factors, such as the high proportion of small and medium-sized enterprises in the country, which often have limited resources to invest in comprehensive safety measures. Additionally, economic challenges over the past decade have strained businesses, potentially leading to compromised safety standards and practices [11,36]. While Greece has a comprehensive set of occupational health and safety regulations, enforcement is often weak. Insufficient funding and staffing of regulatory bodies, along with bureaucratic inefficiencies, contribute to poor enforcement. Inspections are infrequent and penalties for non-compliance are often not stringent enough to serve as effective deterrents [8,9].

The most common injuries reported were fractures, sprains, cuts, and bruises. Fractures were particularly prevalent in high-risk sectors like construction and manufacturing [17]. The primary causes of occupational injuries included machinery-related accidents, falls from heights, and manual handling of heavy objects [17]. Machinery-related accidents were notably frequent in manufacturing and construction. Key risk factors identified were lack of safety training, insufficient protective equipment, and poor regulatory enforcement [18].

Several factors contribute to the high prevalence of occupational injuries in Greece. Firstly, the economic crisis that gripped the country from 2009 onwards led to significant budget cuts, including those affecting occupational health and safety programs. Many workers faced increased workloads and longer hours due to staff reductions and increased demands, leading to fatigue, which is a significant risk factor for occupational injuries. Many businesses, struggling to stay afloat, may have deprioritized safety investments, resulting in outdated equipment and inadequate safety training for employees. The financial crisis led to budget cuts in public health services, reducing access to occupational health services, including preventive care and treatment for work-related injuries [11]. An analysis by Tatsaki et al. for the period between 2000 and 2013 showed a positive correlation between occupational accidents and sanctions imposed by Greek Occupational Safety and Health Inspectors and a negative correlation between GDP and sanctions [36].

Secondly, the regulatory framework, while robust on paper, faces challenges in enforcement. The lack of stringent oversight allows some businesses to neglect safety protocols without immediate repercussions. Finally, the cultural attitudes towards workplace safety can also play a role. In some cases, there may be a lack of awareness or a dismissive attitude towards the importance of adhering to safety regulations, both among employers and employees. This can lead to unsafe practices becoming normalized, increasing the risk of accidents and injuries. Economic pressures also lead to underreporting of injuries as workers fear job loss or other repercussions [11].

Risk factors for occupational injuries are numerous. Industry-specific risks mean that construction workers, for example, are more likely to experience falls from heights, while healthcare workers face risks from percutaneous injuries and exposure to infectious diseases [16-18,27-29]. Demographic factors show that younger and less experienced workers tend to have higher injury rates, and older workers may suffer more severe injuries due to physical limitations. Behavioral factors include non-compliance with safety protocols and risk-taking behaviors [16-18].

The construction sector is notably hazardous, with high rates of falls from heights, machinery-related accidents, and injuries from manual handling of heavy materials [16,18]. Reported mortality has reached 13% [17]. Young age, lack of experience, and overlooking safety protocols have been reported as risk factors [18,37]. Marhavilas et al. calculated that the most dangerous period for occupational injuries in the Greek construction worksites was the 2000-2003 period and suggested that preventive measures should be implemented at least one year earlier in order to decrease the possibility of fatal accidents [35]. Agricultural workers, on the other hand, face risks from machinery, repetitive tasks, and exposure to chemicals, leading to injuries such as contusions, fractures, sprains, and chronic musculoskeletal conditions. Most accidents are caused by falls on the same level [14]. The use of heavy machinery, non-usage of protective equipment, and the lack of proper training are the main risk factors for farm-related injuries [13-15,38]. Many agricultural workers lack formal training in the safe operation of machinery and handling of animals, leading to a higher incidence of accidents. In rural areas of Greece, occupational injuries are the most common cause of injuries in age group 46-65 years [38].

In the industrial sector, including the power industry and petrochemical industry, contact with moving machinery, hazardous chemicals, heavy lifting, lack of protective equipment, wrong working methods, and working at heights also contribute to the risk of injuries [19-21]. Estimated mortality ranges from 0.3% to 6.25% [19-21]. Additionally, the maritime sector poses unique risks for workers in the shipping and fishing industries, including falls, machinery accidents, and drowning [33,34]. In the military sector, the risk of occupational accidents was associated with age, body fat, time of service in the present unit, specialty, level of education, years of service, and overall military training [22,23]. Healthcare workers, most commonly nurses, often face needle-stick and sharp injuries, which are usually underreported. The most common mechanisms include resheathing of used needles, inappropriate sharps disposal, and garbage collection [26,28,29].

The present review has certain limitations. First, it is limited by the heterogeneity of the included studies and the potential publication bias. Additionally, the variability in reporting standards across studies poses a challenge in synthesizing the data. The studies included in this review displayed notable heterogeneity in terms of study design, data collection methods, and outcome measures. For these reasons, a quantitative analysis was not feasible. Second, we chose to include in the review only studies involving occupational accidents and not chronic occupational diseases. Studies might be based on small sample sizes that do not adequately represent the entire workforce in Greece, potentially limiting the generalizability of the findings. Many studies use a cross-sectional design, which does not allow for examining changes over time or establishing causal relationships. Even though we aimed to include a comprehensive range of studies, the availability of research specifically focused on occupational injuries in Greece was limited. This may be due to the underreporting of injuries or the lack of consistent data collection frameworks in Greek occupational health research, impacting the completeness of our findings.

## Conclusions

Occupational injuries in Greece remain a significant public health issue, with certain sectors being particularly vulnerable. These injuries primarily affect the agricultural, construction, industrial, military, and healthcare sectors. Occupational injuries in Greece are influenced by sector-specific risks, economic pressures, weak regulatory enforcement, informal labor practices, cultural attitudes, inadequate training, psychosocial factors, an aging workforce, seasonal work patterns, and gaps in data and research. Addressing this problem requires a multifaceted approach, including better safety training, proper use of safety equipment, improved regulatory frameworks, and stricter enforcement of existing laws. Future research should focus on longitudinal studies to monitor trends and evaluate the effectiveness of interventions.
